# Supplementary material for: A Francisella tularensis Chitinase Contributes to Bacterial Persistence and Replication in Two Major U.S. Tick Vectors
Source: Pathogens. 2020 Dec 10;9(12):1037. doi: 10.3390/pathogens9121037 (PMC7764610; doi:10.3390/pathogens9121037)
Supplement: Supplementary file 1 [file pathogens-09-01037-s001.pdf]

Supplemental Figures for

A *Francisella tularensis* Chitinase Contributes to Bacterial Persistence and Replication in Two Major U.S. Tick Vectors

Brenden G. Tully and Jason F. Huntley

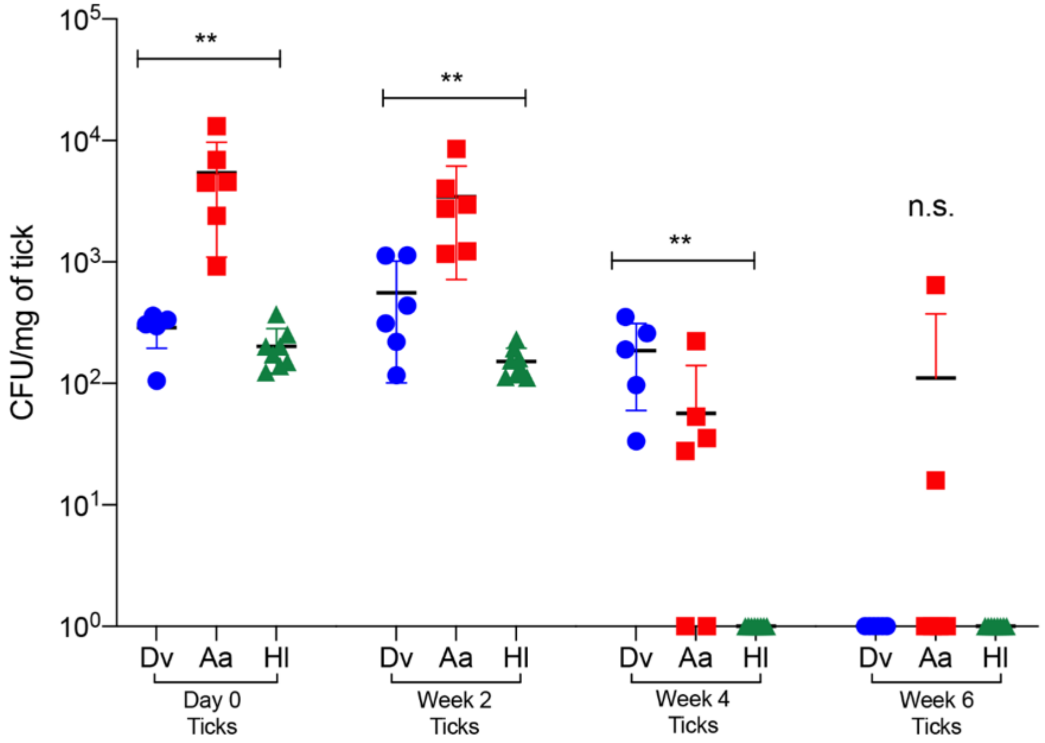

Figure S1. Bacterial numbers (CFU) per mg of tick weight to assess infection, persistence, and replication of *Ft* in ticks after feeding on mice infected with 10^5 CFU. Nymphal *Dv, Aa*, and *Hl* ticks were placed onto non-infected C3H/HeN mice (day -5), ticks fed for 3 days, mice were i.v. infected with 10^5 CFU of *Ft* LVS (day-2), and replete ticks were harvested approx. 2 days later (day 0; 5-day total blood meal). At the indicated time points, individual ticks (n=6-8/tick species) were weighed, homogenized, and plated to enumerate bacterial numbers. CFU/mg of tick were calculated to account for potential differences in *Ft* numbers due to differences in tick weights (e.g., blood meal volume differences or inherent differences in tick weights). One-way ANOVA was used to compare experimental tick groups at each time point: n.s. indicates not significant, ** indicates $P < 0.01$.

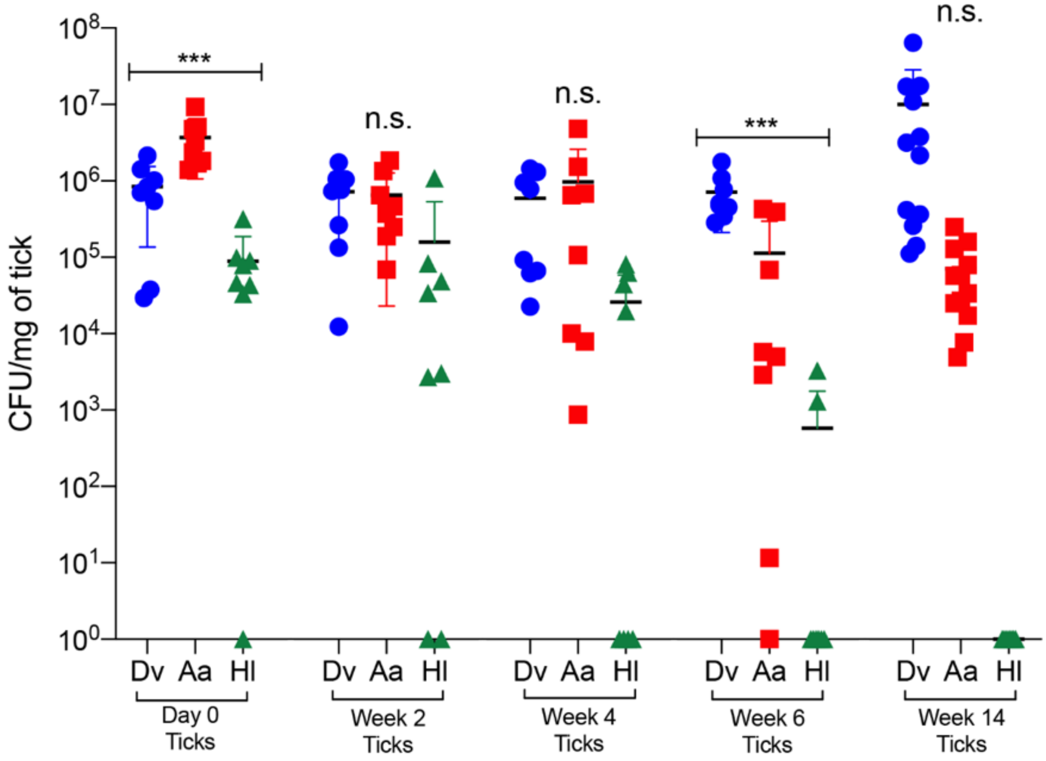

Figure S2. Bacterial numbers (CFU) per mg of tick weight to assess infection, persistence, and replication of *Ft* in ticks after feeding on mice infected with 10^7 CFU. Nymphal *Dv, Aa*, and *Hl* ticks were placed onto non-infected C3H/HeN mice (day -5), ticks fed for 3 days, mice were i.v. infected with 10^7 CFU of *Ft* LVS (day-2), and replete ticks were harvested approx. 2 days later (day 0; 5-day total blood meal). At the indicated time points, individual ticks (n=8-12/tick species) were weighed, homogenized, and plated to enumerate bacterial numbers. CFU/mg of tick were calculated to account for potential differences in *Ft* numbers due to differences in tick weights (e.g., blood meal volume differences or inherent differences in tick weights). One-way ANOVA was used to compare experimental tick groups at each time point: n.s. indicates not significant, *** indicates $P < 0.001$.

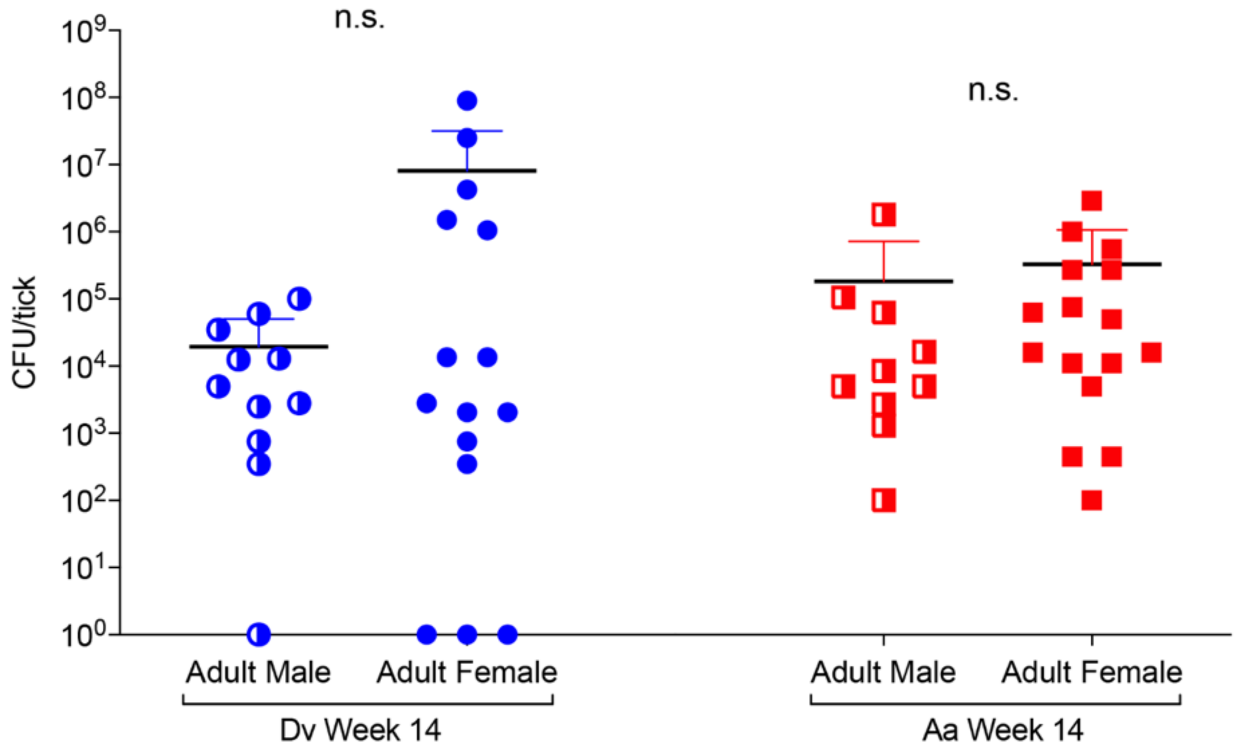

Figure S3. Adult female ticks harbor high *Ft* numbers than adult male ticks. Nymphal *Dv* and *Aa* ticks were placed onto non-infected C3H/HeN mice (day -5), ticks fed for 3 days, mice were i.v. infected with 10^7 CFU of *Ft* LVS (day -2), and replete ticks were harvested approx. 2 days later (5-day total blood meal). Fourteen weeks after completing their blood meal, ticks were homogenized (n=11-16 ticks/sex/tick species) to enumerate bacterial numbers (CFU/tick). Student's *t* test was used to compare male and female experimental groups of ticks of the same species: n.s. indicates not significant.

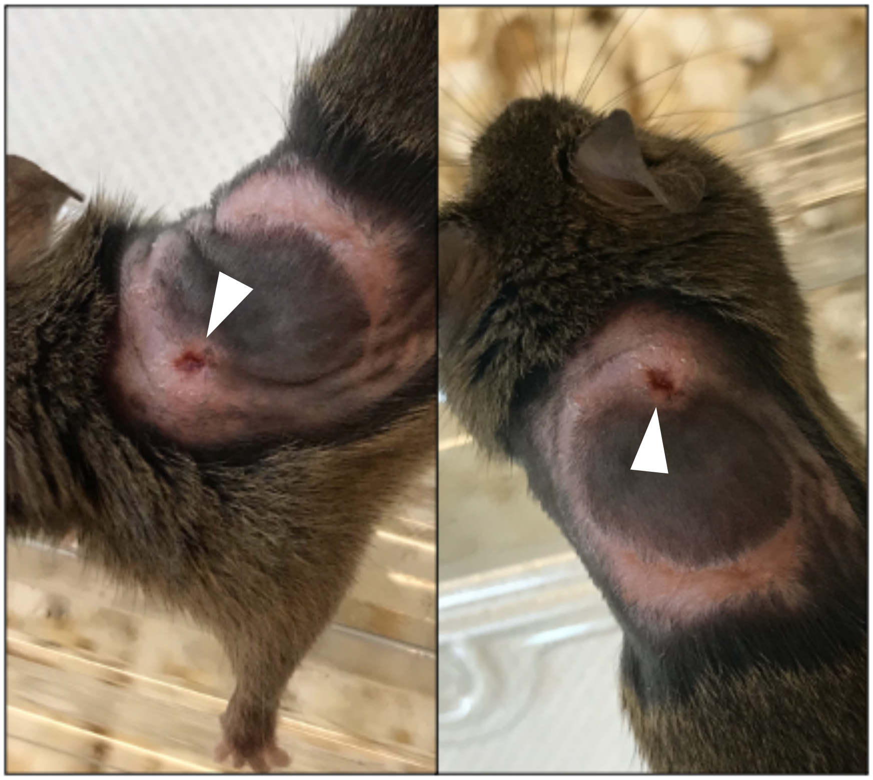

Figure S4. ***Ft*-infected ticks induce skin ulcerations on naive mice.** Infected adult female *Dv* and *Aa* ticks were placed onto naïve mice, together with non-infected adult male ticks of the same species, allowed to feed until repletion (7-12 days), harvested, and mice were monitored until moribund (0-2 days after tick repletion) or euthanized after 21 days. Immediately following tick detachment, tick chambers were removed from the backs of mice and skin ulcers were observed at the tick attachment site in 37% (3 of 8) of *Dv*-infested mice and 33% (2 of 6) of *Aa*-infested mice. No differences in skin ulcerations were observed when comparing *Dv*- and *Aa*-infested mice. Representative skin ulcers from *Dv*-infested mice are shown. White arrowheads point to skin ulcers.

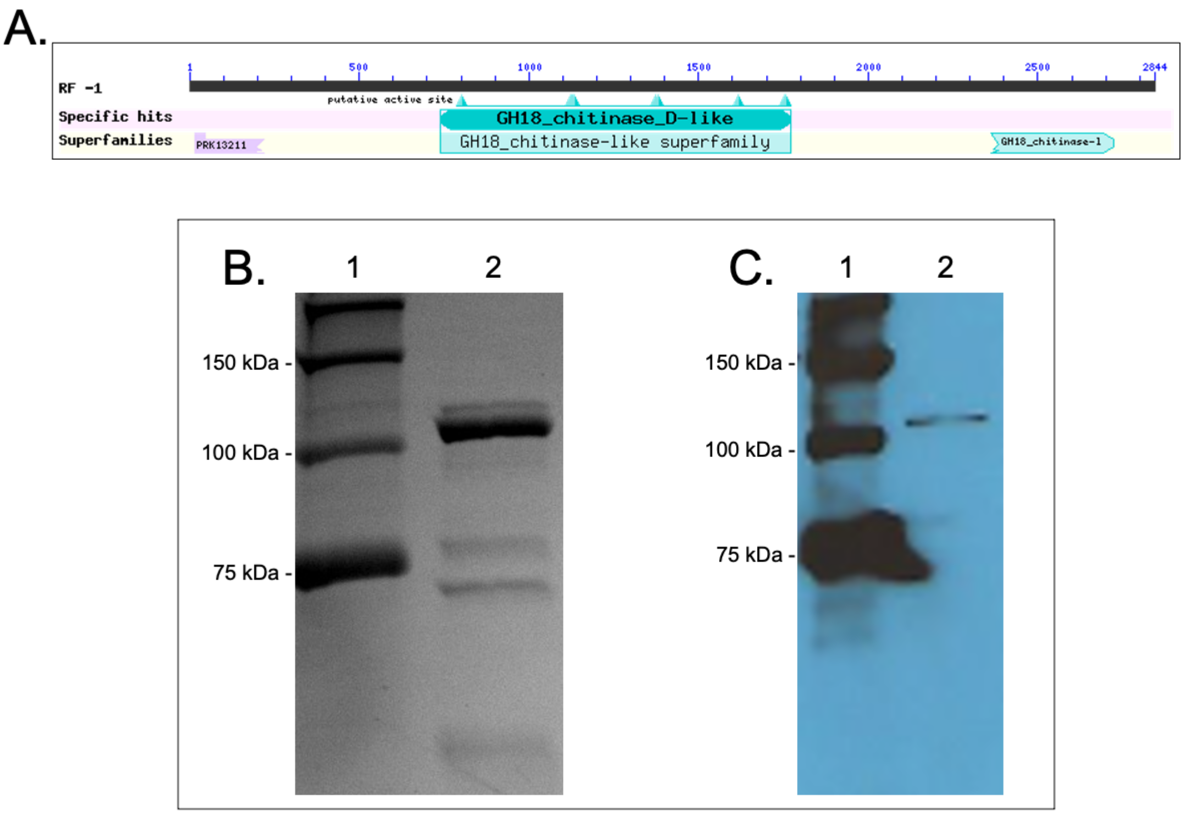

Figure S5. Expression and purification of recombinant FTL1793. (A.) A conserved domain search (https://www.ncbi.nlm.nih.gov/cdd/) of *Ft* gene locus FTL1793 revealed a putative glycosyl hydrolase [GH] 18 chitinase D-like domain. A portion of gene locus *FTL1793*, excluding the amino-terminal signal sequence, was PCR-amplified from *Ft* LVS, cloned into the pBad18 arabinose-inducible expression plasmid (with an carboxy-terminal 6 × histidine fusion tag), transformed into *E. coli* Rosetta bacteria, and recombinant FTL1793 protein was expressed, extracted, and purified by affinity chromatography. The purity of recombinant FTL1793 was assessed by separation on SDS-PAGE followed by: (B.) Coomassie staining; and (C.) transfer to nitrocellulose and immunoblot analysis using a monoclonal antibody specific for the 6 × histidine fusion tag. Lane 1 of each gel is the molecular mass standard with sizes (in kDa) noted on the left of each gel. Lane 2 of each gel is recombinant FTL1793.

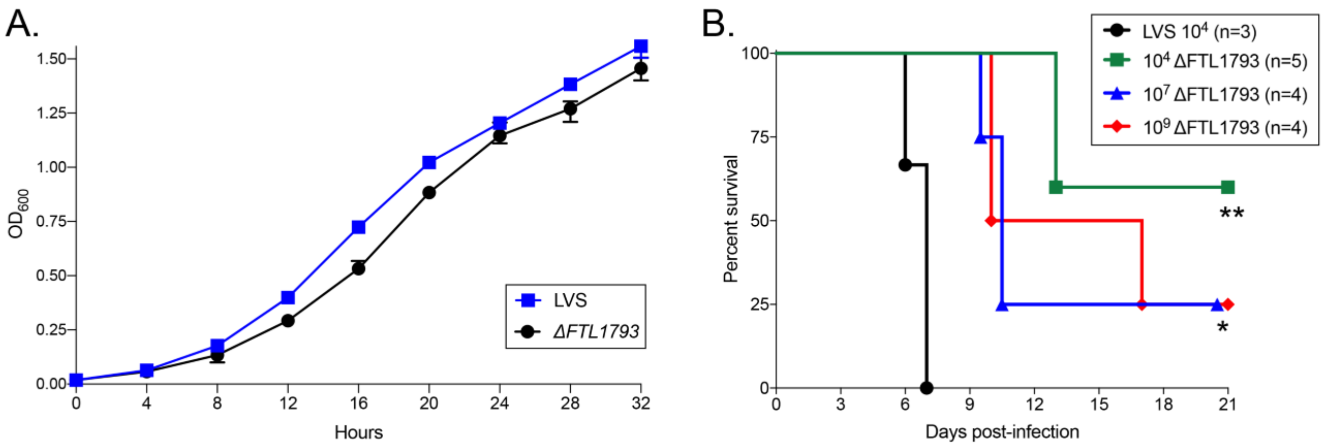

Figure S6. Δ*FTL1793* is partially-attenuated in an intranasal mouse infection model. A gene deletion mutant, Δ*FTL1793*, was generated in *Ft* LVS by homologous recombination. (A.) Growth of wild-type *Ft* LVS and Δ*FTL1793* were compared in supplemented Mueller Hinton medium to assess any inherent growth defects of Δ*FTL1793*. Three independent cultures were inoculated for each strain and growth was monitored by OD_{600} measurements at the indicated time points. (B.) Virulence of Δ*FTL1793* was compared to wild-type LVS in a mouse pulmonary infection model. Groups of C3H/HeN mice were intranasally-infected with either 10^4 CFU of LVS (n=3 mice) or 10^4 CFU (n=5 mice), 10^7 CFU (n=4 mice), or 10^9 CFU (n=4 mice) of Δ*FTL1793*. Mice were monitored through day 21 post-infection. Differences among groups were assessed using the log-rank Mantel-Cox test: * indicates $P < 0.05$; ** indicates $P < 0.01$.
